# Present and projected future mean radiant temperature for three European cities

**DOI:** 10.1007/s00484-017-1332-2

**Published:** 2017-04-26

**Authors:** Sofia Thorsson, David Rayner, Fredrik Lindberg, Ana Monteiro, Lutz Katzschner, Kevin Ka-Lun Lau, Sabrina Campe, Antje Katzschner, Janina Konarska, Shiho Onomura, Sara Velho, Björn Holmer

**Affiliations:** 1 Department of Earth Sciences, University of Gothenburg, Göteborg, Sweden; 2 Department of Geography, Porto University, Institute ISPUP, Porto, Portugal; 3 Department of Architecture and Planning, University of Kassel, Kassel, Germany; 4 Department of Environmental Planning, Brandenburg University of Technology Cottbus-Senftenberg, Cottus, Germany

**Keywords:** Radiant heat load, Climate change, Downtown built-up areas, Building geometry, Trees, Guidelines

## Abstract

Present-day and projected future changes in mean radiant temperature, *T*_mrt_ in one northern, one mid-, and one southern European city (represented by Gothenburg, Frankfurt, and Porto), are presented, and the concept of hot spots is adopted. Air temperature, *T*_*a*_, increased in all cities by 2100, but changes in solar radiation due to changes in cloudiness counterbalanced or exacerbated the effects on *T*_mrt_. The number of days with high *T*_mrt_ in Gothenburg was relatively unchanged at the end of the century (+1 day), whereas it more than doubled in Frankfurt and tripled in Porto. The use of street trees to reduce daytime radiant heat load was analyzed using hot spots to identify where trees could be most beneficial. Hot spots, although varying in intensity and frequency, were generally confined to near sunlit southeast-southwest facing walls, in northeast corner of courtyards, and in open spaces in all three cities. By adding trees in these spaces, the radiant heat load can be reduced, especially in spaces with no or few trees. A set of design principles for reducing the radiant heat load is outlined based on these findings and existing literature.

## Introduction

Exposure to heat may cause severe illnesses and deaths during intense heat events, especially in large urban areas due to the altered urban climate conditions (e.g., Dousset et al. 2011; Gabriel and Endlicher 2011). Elderly, infants and persons with pre-existing cardiovascular, respiratory, or psychiatric diseases and people living in social insulation are especially at risk (e.g., Kovats and Hajat 2008). In addition to health status and demographic and socio-economic factors, regional adaptation to heat effects heat stress risks. For example, heat-related mortality rises from lower apparent temperature (i.e., a measure of relative discomfort due to combined heat and high humidity) in north-continental European cities than in Mediterranean cities (Baccini et al. 2008), as people in north-continental cities have not adapted to heat as they have to cold. Although adverse health effects are most severe during intensive heat events, a large part of the population may suffer from heat stress symptoms also during normal summers (Näyhä et al. 2014). During the last decades, air temperature, *T*_*a*_, has risen and episodes of extreme heat have become more frequent in Europe, and the frequency as well as its effect on human health are projected to progressively increase as a result of climate change (e.g., Fischer and Schär 2010; Kovats et al. 2014); yet, research and policies to manage heat and increase adaptation to heat are in their infancy (Maller and Strengers 2011).

During periods with warm daytime and cool nighttime thermal conditions, such as in normal summers or in the beginning of a heat wave, heat-related deaths among the eldest (ages 80+) are primarily attributable to daytime heat stress (e.g., Rocklöv et al. 2011; Thorsson et al. 2014). This might be explained by the fact that those elderly people who are sensitive to heat stress (e.g., Kovats and Koppe 2005; Åström et al. 2011) may die from heat stroke or exhaustion after a single warm day (Rocklöv et al. 2011). During periods with both warm daytime and nighttime thermal conditions, which often occur during long periods of consecutive hot days, heat-related deaths are primary attributed to the warm nighttime conditions among elderly and other age groups (e.g., Fouillet et al. 2006). During these events, the relative warm nighttime thermal conditions prevent people from recuperating at night from the heat stress experienced during the day (Rocklöv et al. 2011).

In order to mitigate negative effects of intense heat events, it is necessary to identify and predict weather conditions which generate severe heat stress as well as spaces/geometries prone to heat with high accuracy. Furthermore, good knowledge about the efficiency of different strategies to reduce heat stress is important in order to take appropriate actions. During intense heat events, characterized by high *T*_*a*_, high solar radiation, and low wind speed, radiant heating constitutes the largest part of the heat load (heat stress) on humans (e.g., Mayer and Höppe 1987; Mayer et al. 2008), explaining 89% of the variance in thermal perception (Lee et al. 2013). The radiant heat load can be calculated with the mean radiant temperature, *T*_mrt_ (Thorsson et al. 2007). *T*_mrt_ is defined as the “uniform temperature of an imaginary enclosure in which the radiant heat transfer from the human body equals the radiant heat transfer in the actual non-uniform enclosure” (ASHRAE 2001). In other words, it describes the radiative exchange between a person and the environment. *T*_mrt_ is directly influenced by the surface geometry (buildings, vegetation, and topography) and surface materials (albedo, emissivity, etc.), which also makes it useful to identify heat-prone urban geometries and to estimate the efficiency of different strategies to reduce the radiant heat load (Thorsson et al. 2014).

Compared to *T*_*a*_, which is characterized by rather small daytime spatial variations, *T*_mrt_ shows large spatial variations during the day (e.g., Emmanuel and Fernando 2007; Mayer et al. 2008; Thorsson et al. 2011), primarily determined by the shadow pattern generated by buildings and vegetation. During clear and warm summer days, the highest *T*_mrt_ are found near sunlit walls at noon, as a result of high direct and reflected short-wave radiation combined with long-wave radiation emitted from the neighboring surfaces exposed to the sun. On these days and in these locations, *T*_mrt_ may be substantially higher than *T*_*a*_. At night, however, when short-wave radiation is absent, *T*_mrt_ is more or less equal to *T*_*a*_.

There are a number of different strategies that can be applied to reduce radiant heating and thus mitigate outdoor heat stress, such as altering the building geometry (street direction, spacing and width, and building height), altering the surface or building materials, and increasing urban greenery. However, most of the strategies to reduce radiant heating during the day have an adverse effect on the radiant component during the night and vice versa (Andersson-Sköld et al. 2015). For example, increasing the building density reduces the radiant component during the day by increasing shadowing and thus reducing the solar radiation at street level and the convective heat transfer from sunlit building and ground surfaces, while it keeps the radiant component relatively high during the night by reducing the escape of long-wave radiation and thus the cooling of air and surfaces (e.g., Holmer et al. 2007; Mayer et al. 2008; Lindberg et al. 2013). The use of “cool” building materials (i.e., high albedo surfaces with low thermal admittance) has only a minor impact on outdoor heat stress (Erell et al. 2014).

One of the few solutions that mitigate both daytime and nighttime outdoor heat stress is vegetation. During the day vegetation—and especially trees—efficiently reduce the radiant component (Ali-Toudert and Mayer 2007a; Lindberg and Grimmond 2011; Konarska et al. 2014) by blocking the solar radiation at ground level (e.g., Heisler 1986; Konarska et al. 2014) and reducing the surface temperature of neighboring shaded surfaces (e.g., Shashua-Bar et al. 2009). Vegetation also cools its nearby surroundings through transpiration (e.g., Rahman et al. 2011; Shashua-Bar et al. 2011; Konarska et al. 2016). However, the location of greenery, as well as species choice, shape, and amount of vegetation, must be considered in order to optimize the benefits derived from urban greenery and prevent potential side effects.

In this paper, present and projected changes in *T*_mrt_ and the locations of hot spots are analyzed for representative examples of built-up areas for three European cities. Furthermore, the use of street trees to reduce radiant heat load during intense heat events (days with high *T*_*a*_, clear skies, and low wind speed) is analyzed, and the concept of hot spots is used to identify where trees are most beneficial. Based on the findings along with existing literature, a set of design principles to reduce radiant heat load at local level is outlined.

## Study area

The cities of Gothenburg, Sweden; Frankfurt, Germany; and Porto, Portugal—representing one northern, one mid-, and one southern European city—were selected for this case study. Gothenburg, located on the Swedish west coast (57° 42′ N, 11° 59′ E), has a marine west coast climate, with relatively cool summers for its latitude (average *T*_*a*_ of 16.3 °C during June to August, 1960–1990) and because of the moderating influence of the warm Gulf Stream relatively mild winters (average *T*_*a*_ of −0.4 °C in December to February). Frankfurt, located in the south-western part of Germany (51° 07′ N, 8° 41′ E), has a temperate-oceanic climate with average summer and winter *T*_*a*_ of 18.6 and 2.2 °C, respectively. Due to its more inland location, it is slightly drier than Gothenburg. Porto is located on the Portuguese west coast (41° 08′ N, 08° 40′ W). The city has a Mediterranean climate with dry and warm summers (19.3 °C) and rainy and mild winters (9.5 °C).

**Fig. 1:**
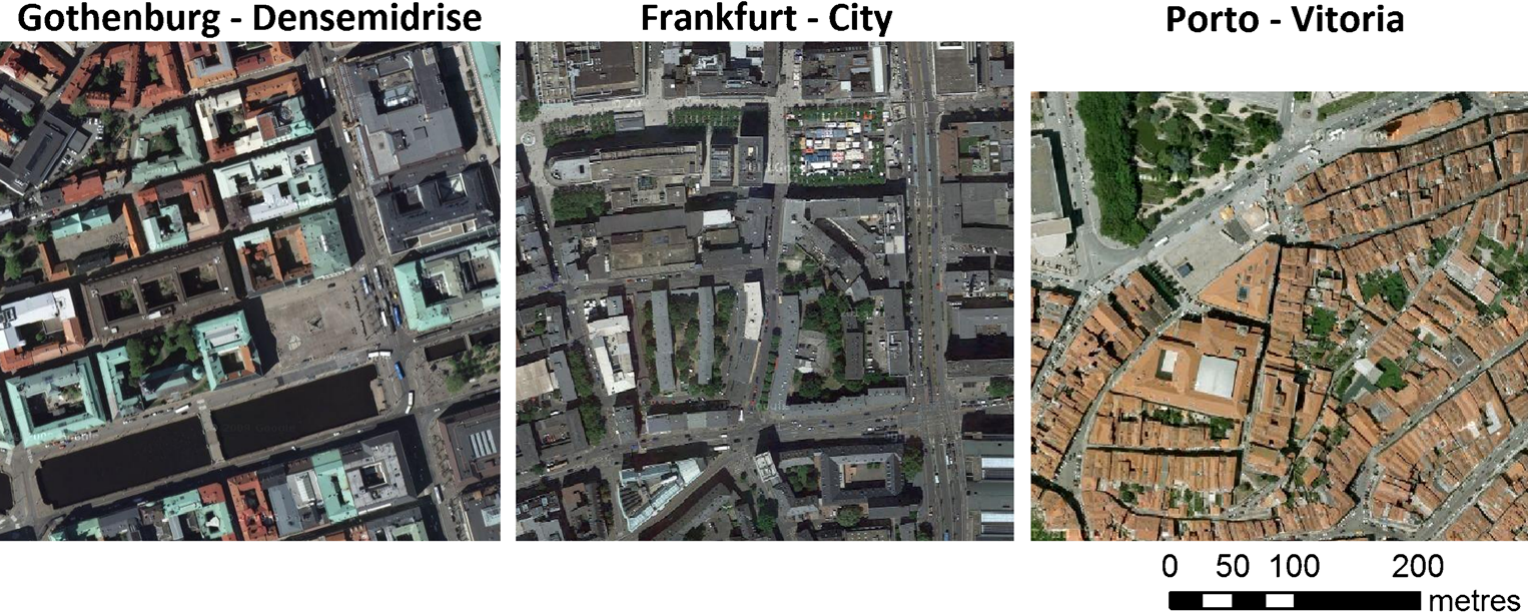
Arial photographs of the three compact mid-rise case study areas

Typical downtown built-up areas characterized by a relatively high building density and little vegetation in each city were selected for case studies (Fig. 1 and Table 1). The building geometries vary across the three cities, from more low- to mid-rise building geometries with wide streets and few street trees in the Gothenburg (allowing solar radiation to reach streets and into the buildings), via a more mid-rise building geometry with some street trees in Frankfurt (creating shade and preventing heat stress in summer) to the low- to mid-rise building geometries with narrow and winding streets (creating shade and preventing heat stress in summer) with limited room for street trees in the more historical neighborhoods in Porto.

**Table 1:** Average building density, building height, street width, tree crown density (buildings excluded), and number of trees per ha ground surface (i.e., buildings excluded) for the three case study areas (compact mid-rise)

City	Average building density (%)	Average building height (m)	Average street width (m)	Tree crown density (%)	Number of trees per ha ground surface
Gothenburg	50	16	24	6.0	21
Frankfurt	48	38	23	8.5	43
Porto	31	15	12	2.7	4

### Meteorological data

Hourly meteorological data, including records of *T*_*a*_, solar radiation (global and diffuse components), and relative humidity, were obtained for each city (Table 2). The observation period used for Gothenburg is 1998–2005, because solar radiation records were interrupted in 2005.

**Table 2:** Description of the meteorological stations and observation periods used

City	Observation period	Land use	Distance from the city center (km)	Meter above sea level City/station	Operated by
Gothenburg	1998–2005	Urban	1.5 (east)	3/5	Swedish Meteorological and Hydrological Institute
Frankfurt	2003–2010	Industrial	10 (west)	100/104	Hessische Landesanstalt für Umwelt und Geologie
Porto	2003–2010	Airport	15 (north-west)	75/69	Instituto Português do Mar e da Atmosfera

### Climate scenarios

Hourly climate scenario time series were created by combining hourly meteorological observations with daily climate model outputs using the method of Rayner et al. (2014). With this method, change factors are first calculated from differences in ranked daily climate model outputs for a future period and a present day, using model time series extracted for a single grid point at the location of each city. Changes consistent with these daily factors are then applied to historical hourly meteorological observations to create hourly future scenarios. A summary of the Rayner et al. (2014) method, based on the description from Lau et al. (2015), is included below.

Climate scenarios were created for each of the three cities for two future periods—mid-century (2040–2069) and end of century (2070–2099). The scenarios used here are based on the fifth-generation atmospheric general circulation model (ECHAM5)/MPI-OM global climate model (GCM) (Roeckner et al. 2003) forced with the Special Report on Emissions Scenarios (SRES) A1B greenhouse-gas emission scenario, downscaled to 25-km resolution with the Rossby Centre Regional Atmospheric Model (RCA3; Kjellström et al. 2005). The SRES A1 scenario family describes rapid, global economic development and is thought to be the type of scenario “best represented” in academic literature when the SRES scenarios were compiled (Nakicenovic and Swart 2000). The A1B scenario, in which the A1 storyline develops using a balance of fossil and non-fossil energy sources, corresponds to a “mid-range” reference scenario (that is, a scenario without deliberate greenhouse-gas mitigation actions), in the context of more recent scenario modeling (Moss et al. 2010). Outputs from regional climate models are widely used for climate-change impact assessments because they simulate finer-scale climatic patterns more realistically than GCMs (e.g.,, Perkins et al. 2014).

Following the algorithm of Rayner et al. (2014), hourly *T*_*a*_ scenarios were created by interpolating the change factors for daily maximum and minimum *T*_*a*_. That is, for each day in the historical record, a change factor for daily maximum *T*_*a*_ was determined from the ranked changes in the regional climate model (RCM) maximum *T*_*a*_ outputs. Change factors for minimum *T*_*a*_ for each day were similarly determined. These series of change factors were then combined, and change factors for every hourly *T*_*a*_ value in the historical record derived using linear interpolation.

For direct and diffuse radiation, change factors for daily global radiation were first calculated for each day in the historical record based on differences in ranked daily short-wave downwelling radiation in the RCM simulations. The short-wave radiation change factor for a given day was applied to all hourly global radiation values for that day. Then, to calculate change factors for the hourly diffuse radiation values, the diffuse radiation was estimated from the global radiation using the method of Reindl et al. (1990), for both the observed and future hourly global radiation. The ratio of these estimates is the change factor that is applied to the observed historical diffuse radiation. Finally, the direct radiation component for the future scenario was determined from the future global and diffuse radiation components. This method ensures that the three radiation components of the scenario time series remain mutually consistent.

Estimation of *T*_mrt_ in Solar and LongWave Environmental Irradiance Geometry (SOLWEIG) model (see below) is most sensitive to short-wave radiation and long-wave radiation calculated via *T*_*a*_, whereas it is almost unaffected by air humidity (Onomura et al. 2015). Thus, the climate scenarios use the unmodified observed hourly relative humidity.

### Spatial and temporal modeling of mean radiant temperature

Present and future spatial variations of *T*_mrt_ within the selected case study areas were calculated using the SOLWEIG model (version 2013a; Lindberg et al. 2008; Lindberg and Grimmond 2011). The model requires hourly weather observations (i.e., *T*_*a*_, air humidity, global and diffuse solar radiation) together with spatial data in form of a digital surface model (DSM) and a geographical location (i.e., latitude, longitude, and altitude). *T*_mrt_ is calculated for a standing or walking person where the angular factors (proportion of radiation received by the human body in each direction) are set to 0.22 for radiation fluxes from the four cardinal points (east, west, north, and south) and 0.06 for radiation fluxes from above and below. Standard values of absorption coefficients for short-wave and long-wave radiation are set to 0.7 and 0.97, respectively (Höppe 1992). Values for albedo and emissivity for buildings and vegetation are set to 0.20 and 0.95, respectively, according to Oke (1987). The transmissivity of short-wave and long-wave radiation through vegetation is set to 5 and 0%, respectively, according to Lindberg and Grimmond (2011). To estimate daytime incoming long-wave radiation (L↓), the approach developed by Crawford and Duchon (1999) is used including fractional cloud cover and *T*_*a*_. Since nighttime sky conditions (i.e., cloudiness) are unknown, we estimate nocturnal L↓ using a simple approach based on Offerle et al. (2003), where the closest daytime value of the sky conditions is used. Thus, nights when a change of weather occurs could affect the estimation of L↓. However, L↓ is in general the smallest of the long-wave fluxes and will therefore have a minor effect on *T*_mrt_ during night. Evaluation of the SOLWEIG model has shown good agreement between modeled and measured radiation fluxes, with the model explaining 94% of the variations in *T*_mrt_, with an overall RMSE of 4.8 K (Lindberg et al. 2008). For a detailed description and evaluation of the SOLWEIG model, see Lindberg et al. (2008) and Lindberg and Grimmond (2011).

In order to study regional differences in *T*_mrt_ with respect to future climate change, *T*_mrt_ for a non-specific (generic) sunlit urban location was calculated using SOLWEIG1D (Lindberg 2012). Unlike the SOLWEIG model described above, where sky view factor (SVF) and shadow patterns are determined for each pixel in a DSM, SOLWEIG1D has a single, fixed, user-specified SVF and the location is assumed to be sunlit during the daytime hours. The latter would not be the case in a real-world situation, where surrounding objects would block the sun at specific times of the day and year when SVF <1. Otherwise, the same settings are used as presented. For these calculations, SVF was set to 0.60.

### Classification of severe heat events and hot spots

Here, *T*_mrt_ ≥60 °C is used to represent severe heat events. The level corresponds to a physiological equivalent temperature, PET >40 °C (Eq. 2 in Lee et al. 2013), i.e., hot thermal conditions (Holst and Mayer 2010). It should be noted that the same thermal conditions might be perceived differently and have different health impacts in the three cities due acclimatization and adaptation to heat (Baccini et al. 2008). However, in order to be able to make cross-European comparison, the same threshold value of *T*_mrt_ for severe heat was used in all three cities. Furthermore, it should be noted that health impact, such as heat-related mortality, might occur at lower levels of *T*_mrt_ (Thorsson et al. 2014).

SOLWEIG1D was used to identify severe heat events, and then, the full version of the model was used to identify the location of hot spots (“Urban geometries prone to heat” section). The method used to identify hot spots, introduced by Lindberg et al. (2016), is to select the highest (90th percentile) values of *T*_mrt_ when *T*_mrt_ at the generic site is ≥60 °C and average these to create “hot spot” maps. All other pixels are set to zero. The occasions when pixels are set to zeroes are included in order to compare the pixels throughout the time period investigated. At the end of a SOLWEIG run, an average of all the maps is calculated. The values in hot spot maps are not actual values of *T*_mrt_ and should be interpreted as an ordinal scale (i.e., hot, hotter, hottest).

## Results and discussion

### Regional climate change

Here, the observed and projected future changes in *T*_*a*_, solar radiation, and *T*_mrt_ across the annual cycle, but focusing on the summer season, are presented and discussed for the three cities.

#### Air temperature

**Fig. 2:**
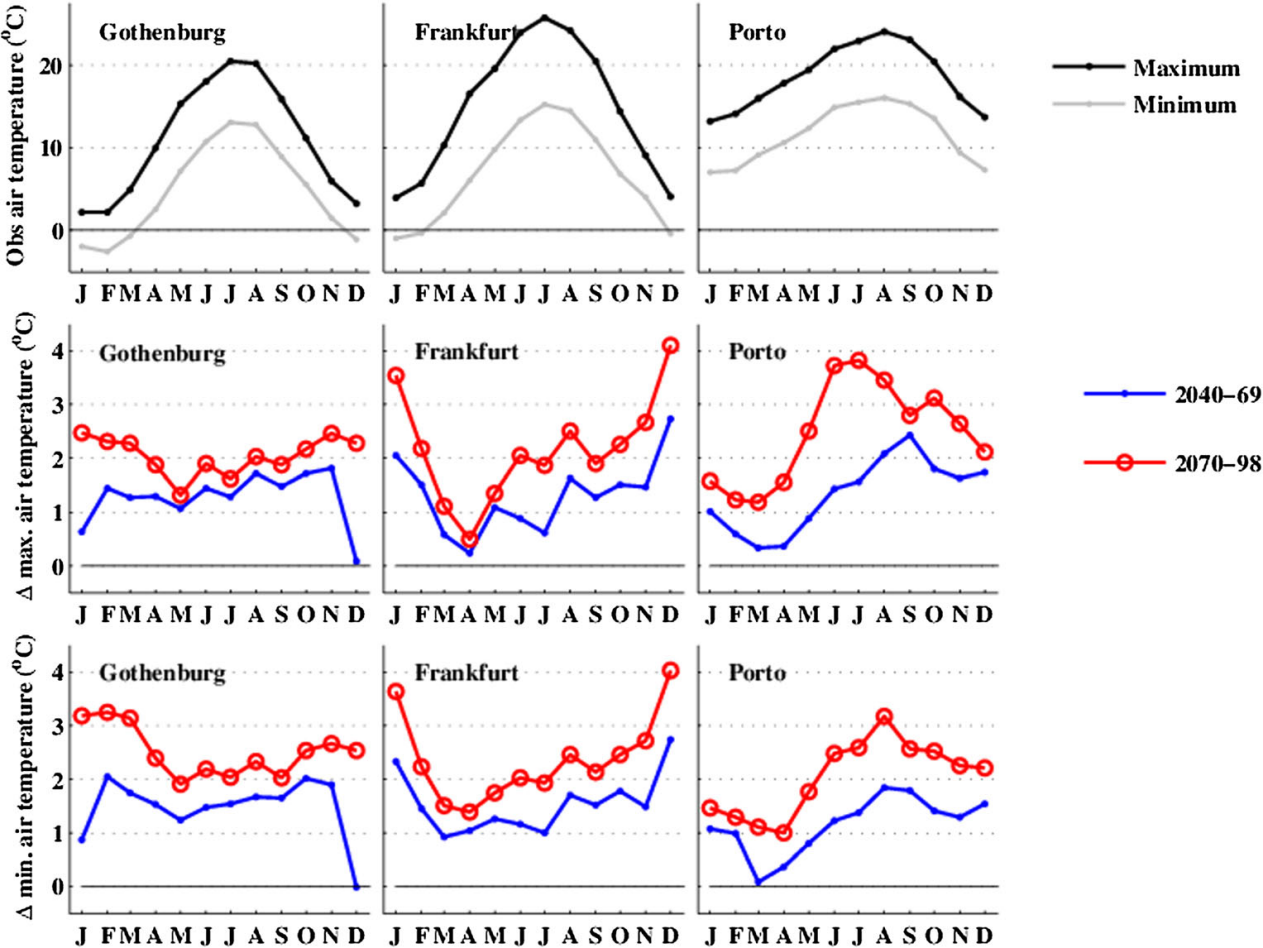
*top row* Observed annual cycle of daily maximum and minimum air temperature, *T*_*a*_, for the three cities. *middle row* Projected future changes in monthly-averaged daily maximum *T*_*a*_ across the annual cycle over the two time periods (2040–2069 and 2070–2098). *bottom row* Changes in monthly-averaged daily minimum *T*_*a*_

Figure 2 shows the observed monthly-averaged daily maximum and minimum *T*_*a*_, and projected future changes, across the annual cycle for the three cities. In line with previous studies (e.g., Christensen et al. 2007; Fischer and Schär 2010; Kjellström et al. 2013), the downscaled scenarios show progressively increasing *T*_*a*_ (both minimum and maximum) in all three cities by 2100. The magnitude, however, differs between the cities and seasons. In Gothenburg and Frankfurt, daily maximum *T*_*a*_ increases less in summer (June-July-August (JJA)) (+2 and +2.1 °C, respectively) than in winter (DJF) (+2.6 and +3.3 °C, respectively), whereas in Porto, the largest increase takes place in summer (+3.3 °C in summer and +1.6 °C in winter; Fig. 2, middle row).

In Gothenburg, daily minimum *T*_*a*_ (i.e., late night or early morning) increases more than daily maximum *T*_*a*_ by the end of the century (Fig. 2, middle and bottom rows). In Frankfurt, the increases in maximum and minimum *T*_*a*_ are more or less similar. In Porto, increases in average summer daily maximum *T*_*a*_ are higher than for minimum *T*_*a*_, whereas they are similar in the winter.

The changes in monthly-averaged daily maximum *T*_*a*_ by 2070–2098 hide potentially important changes in daily maximum *T*_*a*_ distributions (not shown). In Frankfurt, the increase in *T*_*a*_ of warm (98th percentile) summer days is +4.2 °C, substantially larger than the median increases in maximum *T*_*a*_ (+2.1 °C). In Porto, the median increase in maximum *T*_*a*_ (+3.6 °C) is larger than the increase for cold (2nd percentile) summer days (+2.5 °C). In Gothenburg, the increase in maximum *T*_*a*_ is about the same for cold and warm summer days (1.8 and 2.0 °C for the 2nd and 98th percentiles). The results imply that the cities with already high summer maximum *T*_*a*_ will experience the largest increases on warm days.

#### Solar radiation

**Fig. 3:**
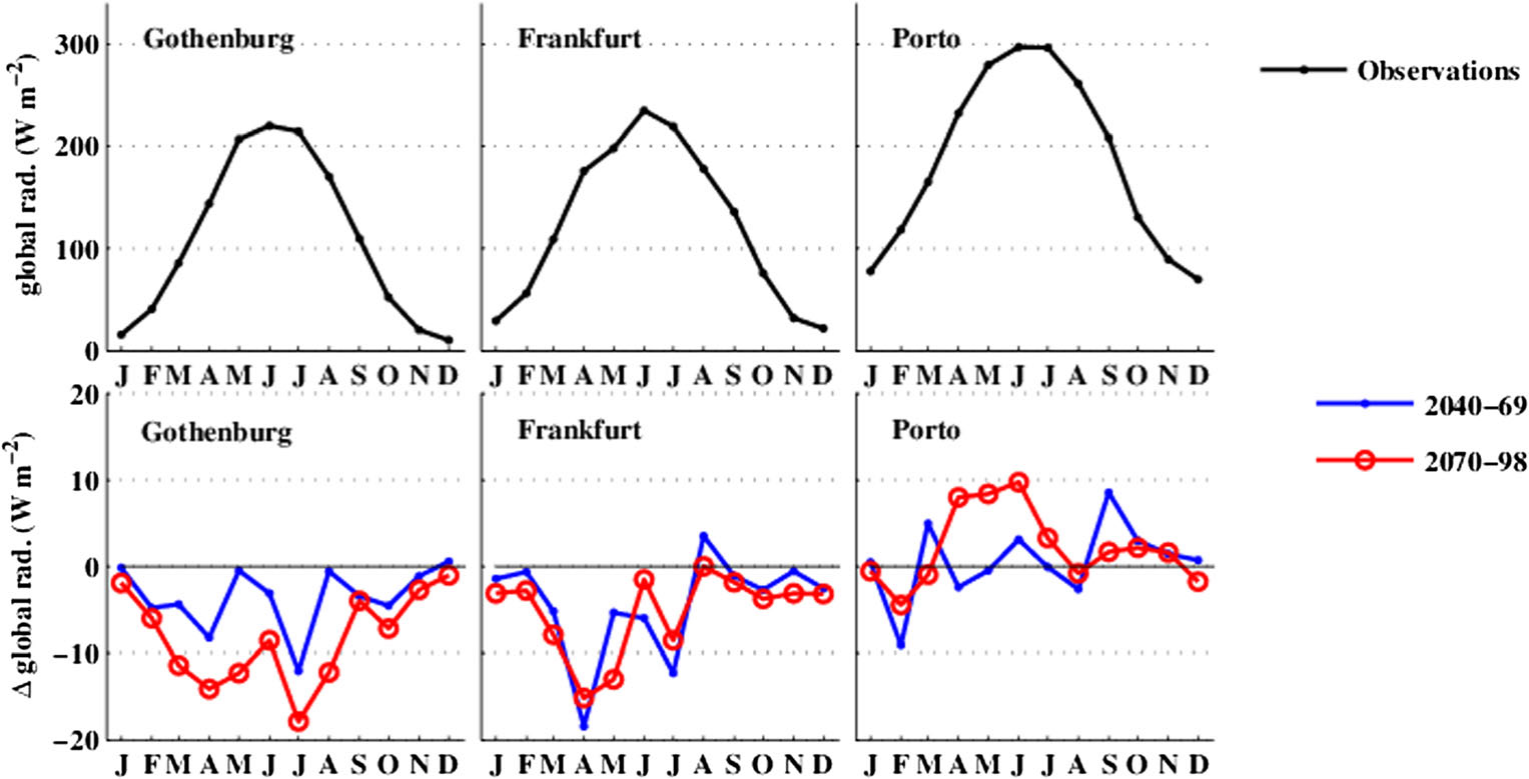
*top row* Observed annual cycle of daily-averaged global solar radiation for the three cities. *bottom row* Projected future changes in daily-averaged global solar radiation across the annual cycle over the two time periods (2040–2069 and 2070–2098)

Figure 3 shows the observed monthly-averaged daily global solar radiation and projected future changes across the annual cycle for the three cities. Solar radiation, which is a function of latitude and time of year, increases further south (in northern hemisphere) and reaches its maximum in summer. Compared to the patterns of change in *T*_*a*_, patterns of change in solar radiation in the downscaled scenarios are much more mixed across the three cities (Fig. 3, bottom row). Daily-averaged global solar radiation in Gothenburg decreases by around 13 Wm^−2^ in summer (JJA) by 2100 due to increase in cloudiness. A smaller decrease occurs in Frankfurt (−3 Wm^−2^). Again, these average changes hide potentially important changes in distributions, with 98th percentile hourly global solar radiation in summer decreasing by 29 Wm^−2^ in Gothenburg but increasing by 3 Wm^−2^ in Frankfurt. In Porto, solar radiation increases in summer by the end of this century as cloudiness decreases.

#### Mean radiant temperature

**Fig. 4:**
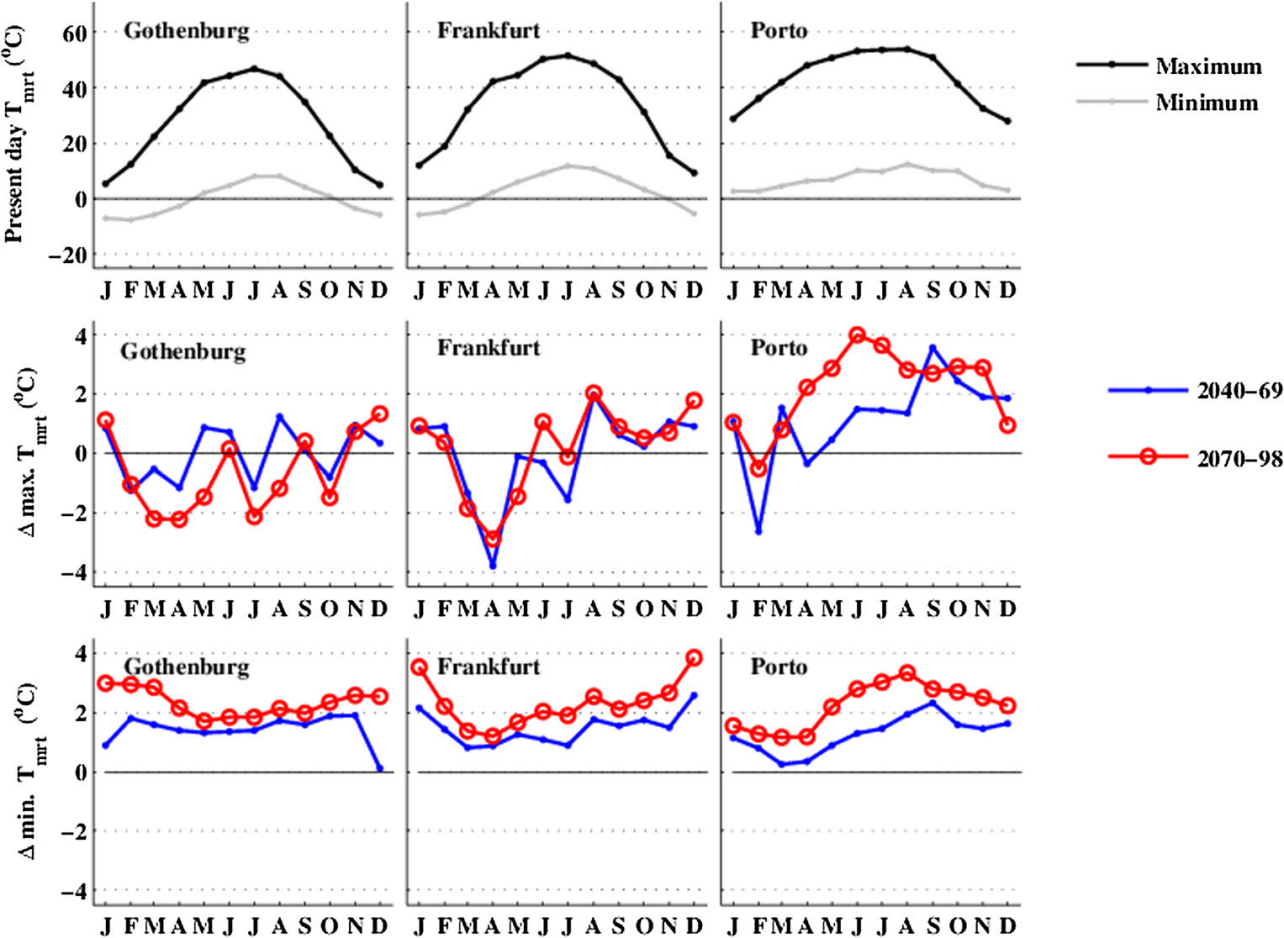
*top row* Calculated annual cycle of daily maximum and minimum mean radiant temperature, *T*_mrt_, for the three cities. *second top row* Projected future changes in monthly-averaged daily maximum, *T*_mrt_, across the annual cycle over the two time periods (2040–2069 and 2070–2098). *third top row* Changes in monthly-averaged daily minimum *T*_mrt_

Monthly average daily maximum and minimum *T*_mrt_ and projected future changes across the annual cycle for the three cities are shown in Fig. 4. Although changes in *T*_mrt_ are dominated by changes in long-wave radiation via an increase in *T*_*a*_ in the future climate scenarios (Lau et al. 2015), changes in solar radiation may counterbalance or add to the effect on increased *T*_*a*_. For example, in Gothenburg, the projected decrease in solar radiation in summer (Fig. 3, bottom row) will counterbalance the effect of increased *T*_*a*_ (Fig. 2, middle row), keeping the average summer maximum *T*_mrt_ relatively unchanged at ∼45 °C. Average summer daily minimum *T*_mrt_ increases in all three cities (Fig. 4, bottom row), with a larger increase in summer in Porto, compared to Frankfurt and Gothenburg.

**Fig. 5:**
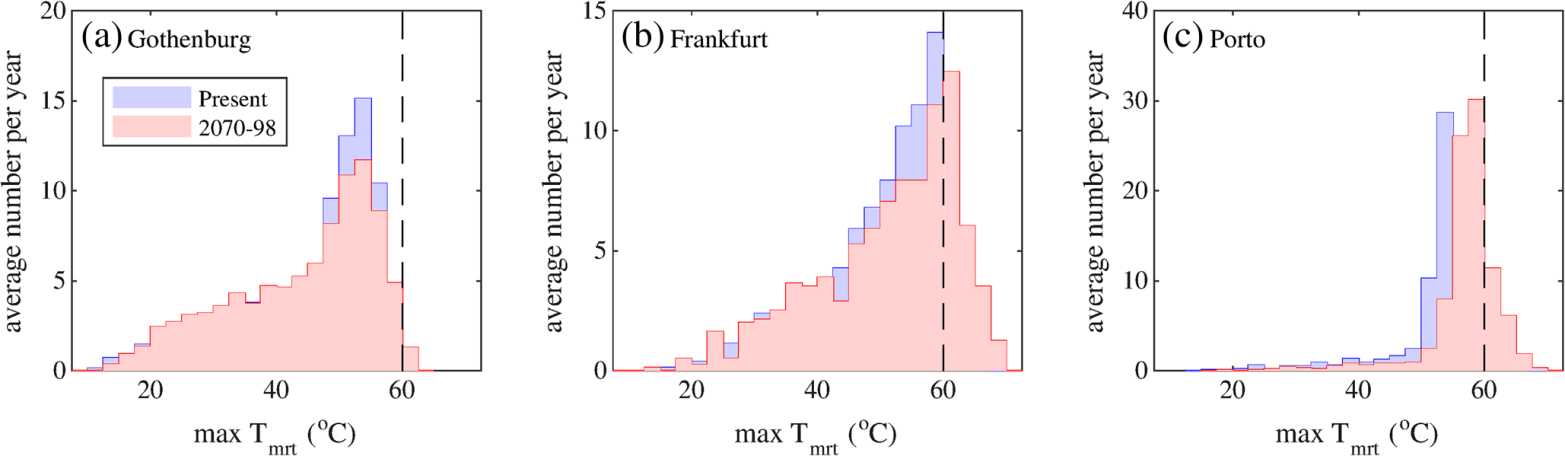
Distribution of daily maximum mean radiant temperature for present day (*blue*) and 2070–2098 (*red*) for the three cities. The *dashed vertical line* indicates the threshold value used to represent severe heat events, *T*_mrt_ = 60 °C

Days with *T*_mrt_ ≥60 °C occur in all three cities in the present climate (Table 3), although to a much lesser extent in Gothenburg than in Frankfurt and Porto. The frequency of such days increases in the downscaled scenario (Table 3), essentially doubling by 2100 in Frankfurt and tripling in Porto, whereas it is relatively unchanged in Gothenburg (+1 day). The average number of days per year in consecutive sequences (two or more days) of *T*_mrt_ >60 °C also increases. The large increase in the average number of days per year with *T*_mrt_ ≥60 °C in Frankfurt relative to Gothenburg can be explained by three factors. As mentioned above, summer daily maximum *T*_*a*_ increases more on hot days in Frankfurt than on average summer days, which is not the case for Gothenburg. Secondly, typical summer maximum hourly global solar radiation in Gothenburg is lower in the future scenario, unlike in Frankfurt. Thirdly, many summer days in Frankfurt have daily maximum *T*_mrt_ very close to 60 °C in the present climate (Fig. 5), so even small increases in *T*_mrt_ can cause relatively large increases in the number of days with *T*_mrt_ ≥60 °C. The highest daily *T*_mrt_ also increases towards the south by the end of the century (Fig. 5).

**Table 3:** Present and projected future changes in (a) average number of days per year with mean radiant temperature, *T*_mrt_ >60 °C in the three cities, and (b) average number of days per year in consecutive sequences (two or more days) of *T*_mrt_ >60 °C

City	(a) Average number of days per year			(b) Average number of consecutive days per year		
	Present	2040–2069	2070–2098	Present	2040–2069	2070–2098
Gothenburg	0.6	+0.4	+0.7	0.2	+0.1	+0.3
Frankfurt	13	+4	+13	5	+3	+8
Porto	9	+7	+18	4	+5	+10

This implies that radiant heat load will be exacerbated in cities where heat stress is already common, with expected severe impact on overall health and well-being as a result (assuming that the relation between *T*_mrt_ and mortality does not change; Thorsson et al. 2014). Furthermore, using *T*_mrt_ (which includes the effect of both solar radiation and *T*_*a*_) as a predictor of heat stress implies that climate change has a larger impact in Porto than would be expected by using *T*_*a*_ alone, whereas in Gothenburg, it implies a lower impact. As a result, the spatial pattern of severe heat stress assessed using *T*_*a*_ alone as a predictor (e.g., Fischer and Schär 2010) is modified (e.g., exhibits a larger north-south gradient in latitude) when accounting for the effects of changes in solar radiation.

It should be noted that the land use, distance to city center, and altitude of the meteorological stations differ between the cities (Table 2). As *T*_*a*_ exhibits a strong gradient between different land uses (e.g., urban-rural) at night, the associated health risks might be underestimated in Porto as the station used is located 15 km outside the city center (i.e., not representing the amplifying effects of the urban heat island).

Another limitation is that this study is based on the outputs from one regional climate model simulation from the ENSEMBLES project (ECHAM5/RCA3). A qualitative analysis of the ENSEMBLE simulations for the three cities shows that the changes in high-percentile summer global solar radiation for ECHAM5/RCA3 were near the middle of the range of the simulations, whereas the simulated changes in high-percentile summer maximum *T*_*a*_ were at the lower end of the range for Gothenburg and towards the upper end of the range for Porto. Further work would be required to investigate how *T*_mrt_ in the study sites changes for different climate models and under different greenhouse gas emission scenarios.

### Urban geometries prone to heat

Here, the location of hot spots and the frequency of high *T*_mrt_ (i.e., *T*_mrt_ ≥60 °C) at street level are presented and discussed for the three cities and time periods. The hot spot maps presented here were calculated without vegetation.

#### Location of hot spots

**Fig. 6:**
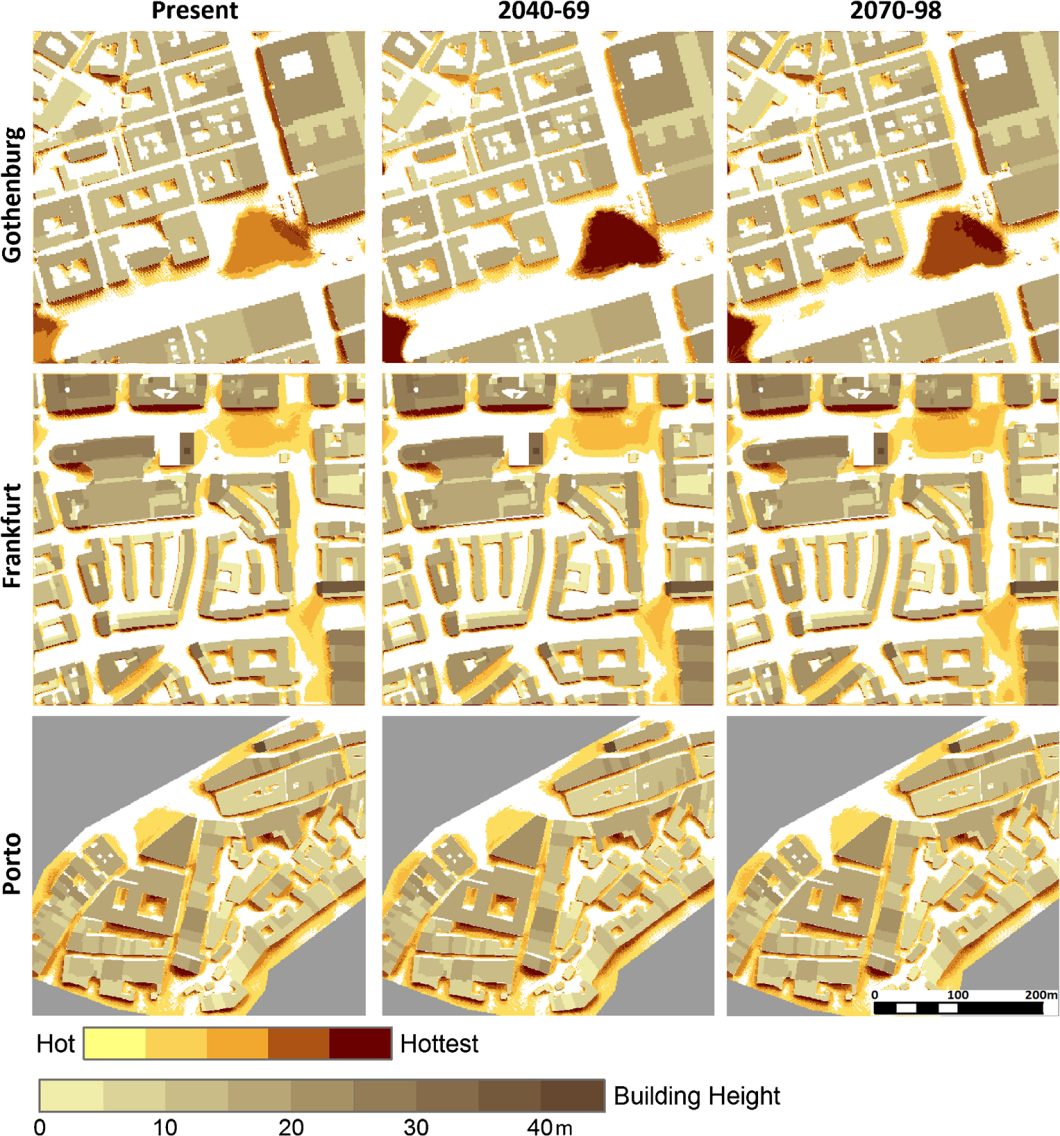
Location of hot spots, graded from hot to hottest spaces, in the three case study areas (without vegetation) over three time periods expressed as 90th percentile mean radiant temperature, *T*_mrt_, at street level when *T*_mrt_ at a generic urban site ≥60 °C

Despite differences in regional climate and climate change, in all three cities and time periods, the hot spots are generally confined to 5–10 m in front of sunlit southeast-southwest facing walls, in the northeast corner of the courtyards, and in open spaces, such as squares and wide streets with relatively high sky view factors (Fig. 6). This is a result of high direct and reflected short-wave radiation fluxes combined with long-wave radiation emitted from Sun-exposed surfaces (e.g., Mayer et al. 2008; Lindberg et al. 2013). Although the general pattern is the same in all three cities and time periods, minor shifts in the location and extension of hot spots occur as a result of differences in solar elevation. For example, hot spots are found in front of east facing walls in Frankfurt, in narrow street canyons with both east-west and north-south directions in Port (except for very close to north facing walls), and at the southwest corners of buildings at street intersections in Frankfurt (height-to-width ratio <2) and Porto (height-to-width ratio >3). On clear summer days, *T*_mrt_ of a sunlit site can be about 30 °C higher than that of an adjacent, shaded street canyon (e.g., Holst and Mayer 2011; Lee et al. 2013). However, when the canyon is lit by the Sun, it can be warmer than the open site, as a result of reflection of long- and short-wave radiation from the surrounding walls (e.g., Thorsson et al. 2011).

For the mid- and end-of-century simulations, some redistribution of the hottest spaces takes place, mainly in Gothenburg but also in Frankfurt. For example, in Gothenburg, spaces in front of west and south facing walls remain as hot spots, although they are no longer the most intense ones. Instead, the most intense hot spots are found in the large open squares. This can be explained by increased cloudiness, which results in reduced surface wall temperature and more intense incoming long-wave radiation from the sky as well as redistribution of short-wave radiation fluxes (Lindberg et al. 2013). Furthermore, spaces in front of east to northeasterly façades show up as being among the hottest spaces by the end of the century, presumably because increased cloudiness during afternoons prevents the west facing façades from heating up to the same extent. In Frankfurt, in particular, the spaces in front of west facing façades and in the northeastern parts of courtyards will no longer be among the most intense hot spots. In Porto, the spatial pattern of hot spots remains relatively stable.

#### Frequency of the occurrence of high *T*_mrt_

**Fig. 7:**
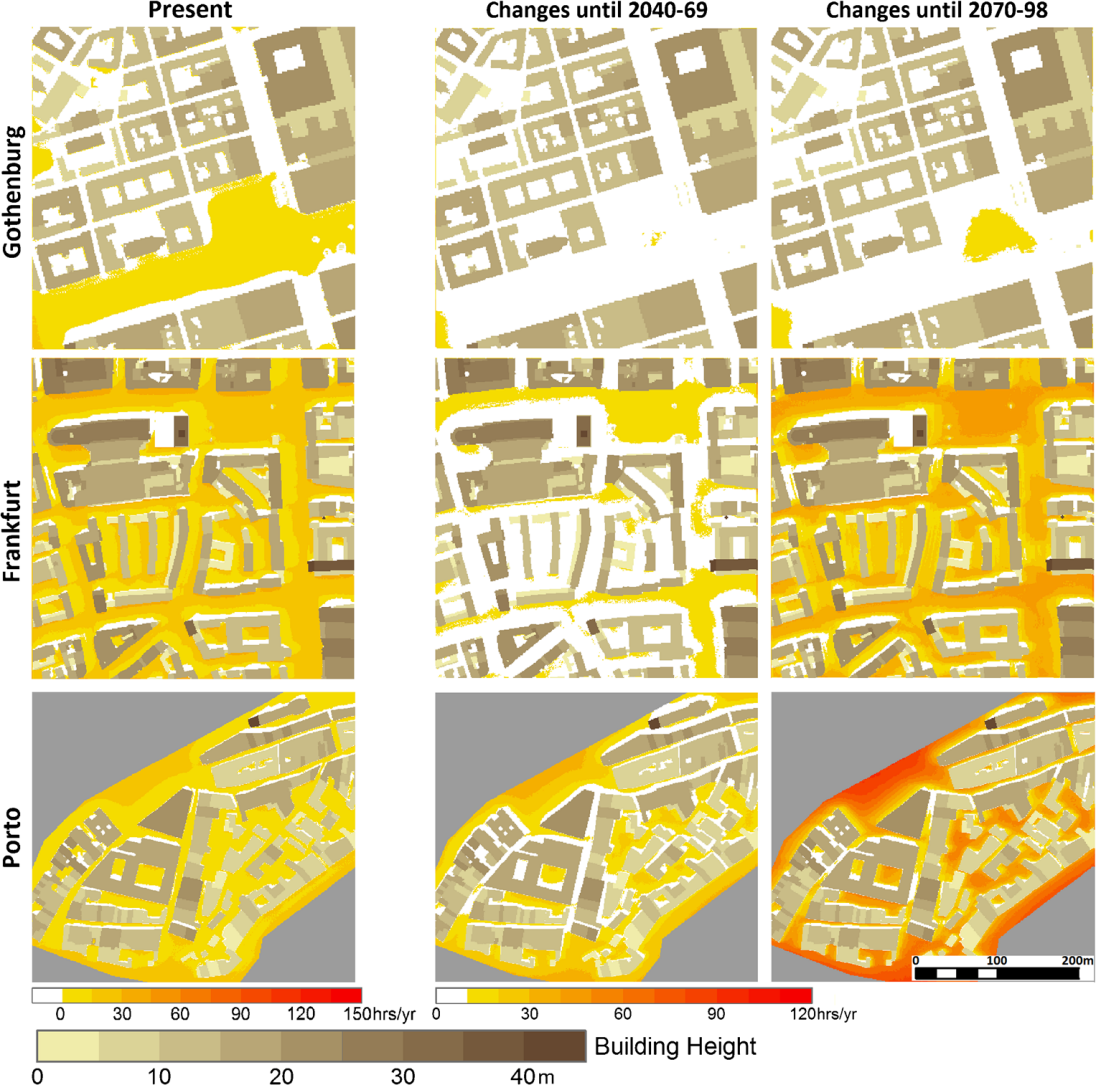
Spatial pattern of the number of hours per year when mean radiant temperature, *T*_mrt_ ≥60 °C, in the three case study areas (without vegetation) over the observation period (*left*). Projected future changes in number of hours by middle and late twenty-first century are shown to the *right*

Figure 7 shows the frequency of the occurrence of *T*_mrt_ ≥60 °C for each pixel in the three study areas over the three time periods. Hours with *T*_mrt_ ≥60 °C occur in large open spaces in all three cities. In Frankfurt and Porto, high *T*_mrt_ also occurs in a majority of the intervening spaces between buildings (courtyards and streets of both directions, i.e., east-west and north-south oriented) as a result of the higher solar elevation. Only pixels in front of north facing walls (<5 m in Frankfurt and <3 m in Porto) are exempt. This concurs with previous findings that although a compact building structure reduces *T*_mrt_, additional measures must be taken to reduce the radiant heat load at lower latitudes (e.g., Ali-Toudert and Mayer 2007b).

It has been shown that in northern and mid-European cities, the orientation of street canyons should be considered in built-up areas with a height-to-width ratio of ≤1 (Lau et al. 2015). In southern European cities, the corresponding value is ≤2. At lower latitudes, high *T*_mrt_ occurs less frequently in north-south oriented street canyons than in east-west oriented canyons because solar access is limited to a few hours during the middle of the day (e.g., Erell and Williamson 2007). Northeast-southwest oriented streets are least favorable in terms of daytime radiant heat load; in that, they experience high *T*_mrt_ because they are less shaded than other streets during the warmest part of the day (Mayer et al. 2008).

In the downscaled scenario, the number of hours of *T*_mrt_ ≥60 °C at street level increases by the middle and end of the century (Table 4), though to a lesser degree in streets and courtyards than in open spaces (Fig. 7). This is in line with previous studies showing that a dense building structure mitigates the impact of a changing climate on outdoor urban *T*_mrt_ (e.g., Thorsson et al. 2011). In Gothenburg and Frankfurt, the average number of hours with *T*_mrt_ ≥60 °C per year is projected to double, whereas in Porto, it is projected to more than triple by 2100 (Table 4). The fraction of street-level space affected by high *T*_mrt_ within the selected study areas will increase in all cities (Fig. 6), from 71 to 75% in Gothenburg, from 92 to 95% in Frankfurt, and from 96 to 98% in Porto by the end of the century. This implies that high *T*_mrt_ will become more frequent and the areas affected will expand in all three cities in the future, with open spaces being most affected. Although a dense building geometry increases shade and reduces daytime heat stress, it cools more slowly at sunset (e.g., Oke 1987; Holmer et al. 2007) and can thus cause thermal discomfort at night. This implies that the spaces that are warmest during the day are not the same spaces that are warmest during the night. According to Lau et al. (2015), a dense building structure does not cause substantial changes in average and minimum *T*_mrt_ in winter.

**Table 4:** Present and projected future changes in (a) average number of hours per year with mean radiant temperature, *T*_mrt_ >60 °C in the three cities, and (b) average number of hours per year in consecutive sequences (two or more days) of *T*_mrt_ >60 °C

City	(a) Average number of hours per year			(b) Average number of consecutive hours per year		
	Present	2040–2069	2070–2098	Present	2040–2069	2070–2098
Gothenburg	0.7	+0.4	+1.1	0.2	+0.2	+0.6
Frankfurt	30	+13	+44	16	+9	+31
Porto	20	+25	+59	10	+16	+39

It should be noted that *T*_mrt_ ≥60 °C can occur at street level even though *T*_mrt_ does not reach this level at our generic urban site, which implies that the number of hours per year with *T*_mrt_ ≥60 °C at street level can be larger than shown in Fig. 7 and Table 4.

### Effect of street trees on mitigating radiant heat load

Here, the concept of hot spots is used to identify where trees are most beneficial. The extent to which the street trees in the study area mitigate radiant heat load is also investigated. Depending on the location and size of the trees, average *T*_mrt_ can be reduced by up to 30 °C and the number of hours with *T*_mrt_ >60 °C at street level can be reduced by up to 40 hours per year, with the largest reduction under and north/northeast of trees located in open, sunlit spaces (not shown). It should be noted that trees in Sun-exposed locations can increase heat stress underneath the tree canopy (Lindberg et al. 2013). This is because vegetation canopies block the relatively colder sky, resulting in increased incoming long-wave radiation originating from vegetation rather than from sky.

It has been shown that trees in open areas—those not shaded by buildings or other trees—reduce *T*_mrt_ more than trees in dense and/or highly wooded sites (e.g., Ali-Toudert and Mayer 2007a; Lindberg et al. 2016). Furthermore, a few large trees provide more shade than many closely spaced small trees (Lau et al. 2016). While the cooling effect of trees is most needed in heat-prone areas, these areas are also stressful environments for trees, which may reduce tree vitality and shorten life span (e.g., Sæbø et al. 2005).

It is important to recognize that creating too much excessive shade—either from trees or buildings—can have unwanted effects. This is especially important in high-latitude cities, which in winter benefit from direct sunshine. Compared to evergreen trees, defoliated deciduous trees allow 40 to 80% solar radiation to penetrate in winter when solar access is desired (e.g., Heisler 1986; Konarska et al. 2014). From this perspective, deciduous trees are more beneficial than evergreen trees in high-latitude cities. Another unwanted effect of street trees is that they can reduce ventilation (e.g., Shashua-Bar et al. 2009).

A set of general design principles for reducing radiant heat load, based on the findings presented and discussed above, is outlined in Appendix A.

## Conclusions

Present and projected future changes in *T*_mrt_ in one northern, one mid-, and one southern European city (represented by Gothenburg, Frankfurt, and Porto) were analyzed. In Gothenburg, the number of days with high *T*_mrt_ is projected to be more or less the same at the end of the century as at present, whereas it will more than double in Frankfurt and triple in Porto. The result implies that the radiant heat load will be exacerbated in the cities where heat stress is already common, while remaining relatively unchanged where heat stress is today a limited problem.

The location of hot spots within typical real-world, built-up areas in each city and the role of street trees in reducing the radiant heat load during heat stress days was also analyzed. The concept of hot spots was adopted to identify where trees are most beneficial. Although varying in intensity, frequency, and spatial extension, hot spots are mainly confined to in front of sunlit, southeast-southwest facing walls; the northeast corner of courtyards; and open spaces such as squares and wide streets with relative high sky view factors. Hot spots also occur in front of east facing walls in Frankfurt, in narrow street canyons of both directions in Porto, and at the southwest corners of buildings at street intersections in Frankfurt and Porto. This remained true also in the future.

Studies today still use *T*_*a*_, sometimes adjusted for humidity, when analyzing the impact of climate and climate change on health. However, changes in both *T*_*a*_ (which influences long-wave radiation) and solar radiation should be considered when analyzing the impact of climate and climate change on people’s health. This is because changes in solar radiation (due to changes in cloudiness) could either counterbalance or aggravate the effect of increased *T*_*a*_. Using *T*_mrt_ or human-biometeorological indices such as PET could give more accurate estimates of the impacts of climate and climate changes on human health than using *T*_*a*_ alone. In some cases, like the one presented here, the spatial pattern of severe heat stress assessed using *T*_*a*_ alone as a predictor might be modified when accounting for the effects of changes in solar radiation.

Since problems with heat stress are expected to increase as a result of climate change, especially in middle and southern Europe, measures to mitigate heat stress must be taken. By increasing the amount of urban greenery, especially by adding trees in sparsely vegetated spaces prone to heat, the incidence of high *T*_mrt_ and thus the magnitude and frequency of heat stress can be reduced. By considering the location of trees as well as the species, shape, and amount of trees, benefits derived from street trees can be enhanced and potential side effects can be prevented.

## Design principles for reducing the radiant heat load in European cities

A set of general design principles for reducing radiant heat load is outlined below. These principles are intended to support urban development and renewal work at local planning levels in regions where a need to minimize outdoor heat stress has been identified. Although the principles are applicable across Europe, local conditions and regional climate peculiarities need to be considered.

Incorporating such design principles into locality’s master or green plans would clearly need to be undertaken with consideration to other issues, such as effects of nighttime and indoor thermal comfort, energy requirements, ventilation of urban street canyons, and urban maintenance. Although we cannot analyze all the complexities, the principles outlined below describe both how the radiant heat load can be reduced and how some important unwanted side effects can be minimized.

The design principles reducing radiant heat load are

- *Increase (and preserve) the amount of urban greenery*
  In order to maximize the cooling effect from greenery
- Trees are to be preferred over lower vegetation, such as bushes and grass as trees provide more shade. Large trees are in turn preferred over small trees.
- Trees should be located in heat-prone areas, i.e., in front of sunlit southeast-southwest facing walls, in northeastern corner of courtyards, and in open spaces with high sky view factors, such as large squares and wide streets as trees in sunlit spaces provides more shade and transpire more. In mid- and low-latitude cities, hot spots also occur in front of east facing walls, in narrow street canyon of both directions, and in the southeast corner of buildings at the street intersections.
- Add trees to open spaces with no or limited trees, as this provides greater heat load reduction than infilling existing tree stands in dense urban spaces.
- Choose species that thrive in relatively warm and dry environments as urban areas are characterized by relatively high air temperature and limited access of water.
  In order to reduce side effects from greenery
- Use urban vegetation to create a mosaic of shaded and sunlit within short distances to enhance and prolong the use of outdoor space.
- Consider deciduous trees over evergreen trees, especially in high-latitude cities, as they give shade in summer but allow solar radiation to penetrate in winter (when solar access is desired).
- Consider vegetation species, location, and shape with a view to minimizing unwanted side effects such as unwanted shade and reduced ventilation.
- *Increase the building density*
  By increasing the building density (i.e., compactness) of urban areas, the spatial extension of hot spots as well as the frequency of high mean radiant temperature can be reduced. In narrow streets, with no room for trees, other types of shading devices should be considered.
  In order to maximize the cooling effect during daytime but allow nighttime cooling, trees can be planted within streets and courtyards. This is especially applicable for low-latitude cities, as much of the street is sunlit during the day due to the high solar elevation.
- *Consider street orientations with respect to building density*
  At height-to-width ratio of ≤1, the orientation of street canyons has a significant impact on street-level heat load in north and mid-European cities. In south European cities, the corresponding value is ≤2. At low latitudes, dense north-south canyons tend to decrease solar access during day and thus the radiant heat load. Northeast-southwest oriented streets have higher heat loads because they provide less shading during the warmest part of the day. At higher height-to-width ratios, street orientation have less effect, but northeast-southwest oriented streets still experience less heat load.
  The unwanted effect of creating too much building shadow can be reduced by considering the height and orientation of buildings. This is especially relevant in high-latitude cities, which benefit from direct sunshine in winter.
- *Use cool building material*

The use of cool building material (i.e., light, reflective surfaces with high albedo, and low thermal admittance) has a minor effect on the radiant heat load at street level. However, light-colored building materials reduce the heat load on buildings and thus the indoor temperature and energy used for cooling and so may be preferred.

## References

[CR1] Ali-Toudert F, Mayer H (2007). Thermal comfort in an east-west oriented street canyon in Freiburg (Germany) under hot summer conditions. Theor Appl Climatol.

[CR2] Ali-Toudert F, Mayer H (2007). Effects of asymmetry, galleries, overhanging façades and vegetation on thermal comfort in urban street canyons. Sol Energy.

[CR3] Andersson-Sköld Y, Thorsson S, Rayner D, Lindberg F, Janhäll S, Jonsson A et al (2015) An integrated method for assessing climate related risks and adaptation alternatives in urban areas. Climate Risk Management 7:31–50

[CR4] ASHRAE (2001). ASHRAE fundamentals handbook 2011, SI Ed.

[CR5] Åström DO, Forsberg B, Rocklöv J (2011). Heat wave impact on morbidity and mortality in the elderly population: a review of 452 recent studies. Maturitas.

[CR6] Baccini M, Biggeri A, Accetta G, Kosatsky T, Katsouyanni K, Analitis A (2008). Heat effects on mortality in 15 European cities. Epidemiology.

[CR7] Christensen JH, Hewitson B, Busuioc A, Chen A, Gao X, Held I, Solomon S, Qin D, Manning M, Chen Z, Marquis M, Averyt KB, Tignor M, Miller HL (2007). Regional climate projections. In: climate change 2007: the physical science basis. Contribution of working group I to the fourth assessment Report of the intergovernmental panel on climate change.

[CR8] Crawford TM, Duchon CE (1999). An improved parameterization for estimating effective atmospheric emissivity for use in calculating daytime downwelling longwave radiation. J Appl Meteorol.

[CR9] Dousset B, Gourmelon F, Laaidi K, Zeghnoun A, Giraudet E, Bretin P (2011). Satellite monitoring of summer heat waves in the Paris metropolitan area. Int J Climatol.

[CR10] Emmanuel R, Fernando HJS (2007). Urban heat islands in humid and arid climates: role of urban form and thermal properties in Colombo, Sri Lanka and Phoenix, USA. Clim Res.

[CR11] Erell E, Williamson T (2007). Intra-urban differences in canopy layer air temperature at a midlatitude city. Int J Climatol.

[CR12] Erell E, Pearlmutter D, Boneh D, Kutiel PB (2014). Effect of high-albedo materials on pedestrian heat stress in urban street canyons. Urban Climate.

[CR13] Fischer EM, Schär C (2010). Consistent geographical pattern of changes in high-impact European heat waves. Nat Geosci.

[CR14] Fouillet A, Rey G, Laurent F, Pavillon G, Bellec S, Guihenneuc-Jouyaux C (2006). Excess mortality related to the August 2003 heat wave in France. Int Arch Occ Env Hea.

[CR15] Gabriel KMA, Endlicher WR (2011). Urban and rural mortality rates during heat waves in Berlin and Brandenburg, Germany. Environ Pollut.

[CR16] Heisler GM (1986). Energy savings with trees. J Arboriculture.

[CR17] Holmer B, Thorsson S, Eliasson I (2007). Cooling rates, sky view factors and the development of intra-urban air temperature differences. Geogr Ann A.

[CR18] Holst J, Mayer H (2010). Urban human-biometeorology: investigations in Freiburg (Germany) on human thermal comfort. Urban Climate News.

[CR19] Holst J, Mayer H (2011). Impact of street design parameters on human-biometeorological variables. Meteorol Z.

[CR20] Höppe P (1992). Ein neues Verfahren zur Bestimmung der mittleren Strahlungstemperatur im Freien. Wetter und Leben.

[CR21] Kjellström E, Bärring L, Gollvik S, Hansson U, Jones C, Samuelsson P (2005). A 140-year simulation of European climate with the new version of the Rossby Centre Regional Atmospheric Climate Model (RCA3).

[CR22] Kjellström E, Thejll P, Rummukainen M, Christensen JH, Boberg F, Christensen OB, Fox Maule C (2013). Emerging regional climate change signals for Europe under varying large-scale circulation conditions. Clim Res.

[CR23] Konarska J, Lindberg F, Larsson A, Thorsson S, Holmer B (2014). Transmissivity of solar radiation through crowns of single urban trees—application for outdoor thermal climate modelling. Theor Appl Climatol.

[CR24] Konarska J, Uddling J, Holmer B, Lutz M, Lindberg F, Pleijel H, Thorsson S (2016). Transpiration of urban trees and its cooling effect in a high latitude city. Int J Biometeorol.

[CR25] Kovats RS, Hajat S (2008). Heat stress and public health: a critical review. Annu Rev Public Health.

[CR26] Kovats RS, Koppe C, Ebi K, Smith J, Burton I (2005). Heat waves past and future impacts on health. Integration of public health with adaptation to climate change: lessons learned and new directions.

[CR27] Kovats RS, Valentini R, Bouwer LM, Georgopoulou E, Jacob D, Martin E, Barros VR, Field CB, Dokken DJ, Mastrandrea MD, Mach KJ, Bilir TE, Chatterjee M, Ebi KL, Estrada YO, Genova RC, Girma B, Kissel ES, Levy AN, MacCracken S, Mastrandrea PR, White LL (2014). Europe. Climate change 2014: impacts, adaptation, and vulnerability. Part B: regional aspects. Contribution of working group II to the fifth assessment Report of the intergovernmental panel on climate change.

[CR28] Lau K, Lindberg F, Thorsson S, Rayner D (2015). The effect of urban geometry on mean radiant temperature under future climate change: a study of three European cities. Int J Biometeorol.

[CR29] Lau K, Lindberg F, Holmer B, Thorsson S (2016) The effect of street geometry design and vegetation on daytime outdoor heat stress: a parametric study based on numerical modelling of mean radiant temperature. Submitted to Build Environ

[CR30] Lee H, Holst J, Mayer H (2013). Modification of human-biometeorologically significant radiation flux densities by shading as local method to mitigate heat stress in summer within urban street canyons. Adv Meteorol.

[CR31] Lindberg F, (2012) The SOLWEIG-model, http://www.gvc.gu.se/Forskning/klimat/stadsklimat/gucg/ software/solweig/, assessed 2014/02/12.2012–06-13: Gothenburg University, Sweden.

[CR32] Lindberg F, Grimmond CSB (2011). The influence of vegetation and building morphology on shadow patterns and mean radiant temperature in urban areas: model development and evaluation. Theor Appl Climatol.

[CR33] Lindberg F, Holmer B, Thorsson S (2008). SOLWEIG 1.0—modeling spatial variations of 3D radiant fluxes and mean radiant temperature in complex urban settings. Int J Biometeorol.

[CR34] Lindberg F, Holmer B, Thorsson S, Rayner D (2013). Characteristics of the mean radiant temperature in high latitude cities—implications for sensitive climate planning applications. Int J Biometeorol.

[CR35] Lindberg F, Thorsson S, Rayner D, Lau K (2016). The impact of urban planning strategies for reducing heat stress in climate change perspective. Sustainable Cities and Society.

[CR36] Maller CJ, Strengers Y (2011). Housing, heat stress and health in a changing climate: promoting the adaptive capacity of vulnerable household, a suggested way forward. Health Promot Int.

[CR37] Mayer H, Höppe P (1987). Thermal comfort of man in different urban environments. Theor Appl Climatol.

[CR38] Mayer H, Holst J, Dostal P, Imbery F, Schindler D (2008). Human thermal comfort in summer within an urban street canyon in central Europe. Meteorol Z.

[CR39] Moss RH, Edmonds JA, Hibbard KA, Manning MR, Rose SK, van Vuuren DP (2010). The next generation of scenarios for climate change research and assessment. Nature.

[CR40] Nakicenovic N, Swart R (2000). Special report on emissions scenarios.

[CR41] Näyhä S, Rintamäki H, Donaldson G, Hassi J, Jousilahti P, Laatikainen T (2014). Heat-related thermal sensation, comfort and symptoms in a northern population: the national FINRISK 2007 study. Eur J Pub Health.

[CR42] Offerle B, Grimmond CSB, Oke TR (2003). Parameterization of net all-wave radiation or urban areas. J Appl Meteorol.

[CR43] Oke TR (1987). Boundary layer climates.

[CR44] Onomura S, Grimmond CSB, Lindberg F, Holmer B, Thorsson S (2015). Meteorological forcing data for urban outdoor thermal comfort models from a coupled convective boundary layer and surface energy balance scheme. Urban Climate.

[CR45] Perkins S, Moise A, Whetton P, Katzfey J (2014). Regional changes of climate extremes over Australia—a comparison of regional dynamical downscaling and global climate model simulations. Int J Climatol.

[CR46] Rahman M, Smith J, Stringer P, Ennos A (2011). Effect of rooting conditions on the growth and cooling ability of *Pyrus calleryana*. Urban For Urban Gree.

[CR47] Rayner D, Lindberg F, Thorsson S, Holmer B (2014). A statistical downscaling algorithm for thermal comfort applications. Theor Appl Climatol.

[CR48] Reindl DT, Beckman WA, Duffie JA (1990). Diffuse fraction correlation. Sol Energy.

[CR49] Rocklöv J, Ebi K, Forsberg B (2011). Mortality related to temperature and persistent extreme temperature: a study of cause-specific and age-stratified mortality. Occup Environ Med.

[CR50] Roeckner E, Bäuml G, Bonaventura L, Brokopf R, Esch M, Giorgetta M et al. (2003) The atmospheric general circulation model ECHAM5, part I: model description. (p. 140). Hamburg

[CR51] Sæbø A, Borzan Z, Ducatillion C, Hatzistathis A, Lagerström T, Supuka J et al. (2005) The selection of plant materials for street trees, park trees and urban woodland. In: Urban forests and trees. Springer, pp 257–280

[CR52] Shashua-Bar L, Pearlmutter D, Erell E (2009). The cooling efficiency of urban landscape strategies in a hot dry climate. Landscape Urban Plan.

[CR53] Shashua-Bar L, Pearlmutter D, Erell E (2011). The influence of trees and grass on outdoor thermal comfort in a hot-arid environment. Int J Climatol.

[CR54] Thorsson S, Lindberg F, Eliasson I, Holmer B (2007). Different methods for estimating the mean radiant temperature in an outdoor setting. Int J Climatol.

[CR55] Thorsson S, Lindberg F, Bjorklund J, Holmer B, Rayner D (2011). Potential changes in outdoor thermal comfort conditions in Gothenburg, Sweden due to climate change: the influence of urban geometry. Int J Climatol.

[CR56] Thorsson S, Rocklöv J, Konarska J, Lindberg F, Holmer B, Dousset B, Rayner D (2014). Mean radiant temperature—a predictor of heat related mortality. Urban Climate.

